# *Hereditary Cancer in Clinical Practice *transfers to BioMed Central

**DOI:** 10.1186/1897-4287-7-1

**Published:** 2009-01-26

**Authors:** Jan Lubiński, Rolf H Sijmons, Rodney J Scott

**Affiliations:** 1 International Hereditary Cancer Center, Pomeranian Medical University, Ul. Polabska 4, 70-115 Szczecin, Poland; 2 Dept. of Genetics, University Medical Center Groningen, University of Groningen, PO Box 30.001, 9700 RB Groningen, The Netherlands; 3 Discipline of Medical Genetics, Hunter Area Pathology Service, John Hunter Hospital, Lookout Road, Newcastle, NSW 2305, Australia

We are proud to announce the re-launch of *Hereditary Cancer in Clinical Practice *(HCCP). Moving from a classical printed journal, first published in 2003, to the open access, online format of BioMed Central will allow for a quicker and much wider distribution of published articles. Further improving exposure of articles, the journal has also been accepted for indexing by PubMed, and is now on track to receive it's first Impact Factor in 2010 after being accepted for inclusion in Thomson Reuters' Science Citation Index Expanded and the Journal Citation Reports. In addition, the Editorial Board of the journal has been expanded in acknowledgement of the continuing growth of the field of clinical cancer genetics.

Much has changed since December 2003 when the first paper issue of *HCCP *was published. New genes and syndromes have been identified, more molecular pathways have been unraveled, and new knowledge on therapeutic and preventive options has been gained. However, the reasons for publishing *HCCP *remain unchanged: to contribute to the improvement of care for individuals and families who are genetically predisposed to develop cancer. As before, this journal will try to cover relevant topics from multiple perspectives, including those of researchers and medical practitioners from different parts of the world who may have different scientific interests and medical priorities. We also remain dedicated to support the international discussion on important topics by sending out questionnaires to the field and publishing their results.

We invite you and your colleagues to submit your manuscripts to *HCCP*, ranging from small but illustrative case reports to larger scientific studies. In addition to these types of articles, the reader may expect to see meeting reports, reviews and commentaries to highlight new developments and existing controversies in the field.

We hope you will enjoy reading the new *Hereditary Cancer in Clinical Practice*.

